# Cardiac Myosin Inhibitors as a Novel Treatment Option for Obstructive Hypertrophic Cardiomyopathy: Addressing the Core of the Matter

**DOI:** 10.1161/JAHA.121.024656

**Published:** 2022-05-03

**Authors:** Ahmad Masri, Iacopo Olivotto

**Affiliations:** ^1^ Division of Cardiology Hypertrophic Cardiomyopathy Center School of Medicine Oregon Health & Science University Portland OR; ^2^ Cardiomyopathy Unit Department of Experimental and Clinical Medicine University of Florence Italy

**Keywords:** aficamten, cardiac myosin inhibitors, hypertrophic cardiomyopathy, mavacamten, myectomy, Cardiomyopathy

The modern perception of hypertrophic cardiomyopathy (HCM), matured over the past 6 decades, is that of a common, genetically diverse, multi‐faceted disease. The meticulous understanding of HCM pathophysiology, combined with creative therapeutic solutions, has allowed us to achieve excellent outcomes at expert centers, particularly in the treatment of patients with symptomatic left ventricular outflow obstruction (LVOTO).^1^ Extended surgical myectomy and alcohol septal ablation (invasive septal reduction therapies—SRT) have solidly become the gold standard treatment in this challenging population, providing symptomatic relief and likely reducing mortality.^2^ Observational literature from expert centers with high surgical volumes converge on the conclusion that obstructive HCM (oHCM) outcomes are excellent with low operative and long‐term mortality.^3^, ^4^, ^5^, ^6^ Nevertheless, many challenges remain ahead and oHCM remains, under many aspects, an orphan condition.

Currently, SRT are largely confined to patients with oHCM who have advanced symptoms, as defined by New York Heart Association (NYHA) Class III or IV.^7^ Increasing evidence suggests that SRT are safe in experienced centers, allowing consideration for patients with NYHA Class II symptoms. However, in a nationwide study of 6386 septal myectomies performed in the United States between 2003 and 2011, 60% of hospitals performed ≤10 procedures over the 9‐year period and in‐hospital mortality was as high as 15.6% in the lowest surgical volume tertile.^8^ Even in the highest tertile, in‐hospital death, need for permanent pacing, stroke, bleeding, and acute renal failure were not negligible (3.8%, 8.9%, 1.9%, 1.7%, and 9.4%, respectively).^8^ Worldwide, many patients with oHCM are managed in institutions with limited or no expertise in SRT.

The initial approach to treatment of symptomatic patients with oHCM includes beta‐blockers (BB), non‐dihydropyridine calcium channel blockers (CCB), and disopyramide.^7^ BB and CCB act as weak negative inotropes and are often insufficient to control symptoms.^9^ In addition, these agents are commonly associated with side effects and chronotropic incompetence. Disopyramide is employed as a second‐line therapy, is safe and often effective in expert hands, but its use is limited by anticholinergic side effects and efficacy may diminish with time, so that SRTs ultimately become necessary. Disopyramide also suffers from supply shortages and lack of confidence by physicians, many of whom are reluctant to use class I anti‐arrhythmic agents in structural heart disease. Notably, none of these time‐honored medications have undergone rigorous evaluation with large multicenter randomized clinical trials (Table).^9^, ^10^, ^11^, ^12^, ^13^, ^14^, ^15^, ^16^ A recent crossover trial showed that BB decrease LVOT gradients and improve symptoms but without improving peak oxygen consumption (pVO_2_).^12^

Importantly, SRTs are effective in palliating symptoms and possibly improving longevity in oHCM patients, but do not address the core mechanisms of disease at the functional and energetic level. Thus, the natural evolution of the cardiomyopathic process is not affected. In a survey‐based study of 753 patients (with a median age at survey of 64 years) undergoing myectomy, 26% reported new onset of atrial fibrillation (AF) during follow‐up, increasing to 37% among those followed over 10 years.^17^ As AF is an important marker of disease progression, and a predictor of adverse outcome in HCM, this observation challenges the notion of myectomy as a definitive treatment. This is consistent with data from the SHARE (Sarcomeric Human Cardiomyopathy Registry) on 4591 patients, showing a substantial and progressive burden of HCM‐related morbidity in both obstructive (28%) and nonobstructive patients (including those undergoing SRT) mainly driven by heart failure and AF.^18^ Thus, in accepting the status quo, we accept the lack of effective disease‐modifying strategies for our patients.

The 2020 American College of Cardiology/American Heart Association HCM guidelines have placed significant emphasis on shared decision‐making in HCM across multiple domains, including treatment approaches.^7^ Diversifying treatment strategies is the best way of empowering patients and promoting personalized care. In the current standard‐of‐care approach, it is assumed that all oHCM patients are willing to undergo SRT, have insurance plans that cover them in seeking care at an experienced center, and are able to afford the cost of travel for themselves and their family members, as well as the time off required for recovery from SRT. Such pre‐requisites to getting excellent care at the few expert centers promote healthcare inequality by inadvertently excluding patients with lower socioeconomic status, lower education level, those who cannot advocate for themselves, and patients living with disabilities.

Over the past 2 decades, the need for more effective and less invasive therapies combined with advances in our understanding of HCM pathophysiology have set the stage for developing new agents targeting the molecular basis of the disease. HCM‐associated mutations affecting sarcomere protein genes have been shown to cause myocardial hyper‐contractility, due to excessive availability of myosin heads ready to form cross‐bridges with actin, with a reduced proportion remaining in the energy‐sparing super‐relaxed state not available for engagement. This is thought to represent the core pathophysiological abnormality ultimately generating the HCM phenotypes, from compensatory hypertrophy to diastolic impairment, from LVOTO to arrhythmias, and from energy depletion to fibrosis. Inhibiting the myosin ATPase via selective cardiac myosin inhibitors (CMI) counters this state of things by reducing the number of myosin heads available for engagement with resultant return to a normal or quasi‐normal contractile state, relief of LVOT obstruction, decrease in wall stress, and improvement in lusitropy.^19^ Currently, there are 2 main CMIs currently in various stages of development, mavacamten and aficamten. In a murine model harboring heterozygous pathogenic mutations in the cardiac myosin heavy chain, chronic administration of mavacamten suppressed the development of ventricular hypertrophy, cardiomyocyte disarray and myocardial fibrosis, and attenuated hypertrophic and profibrotic gene expression.^20^ These potent and protean effects support a disease‐modifying potential for CMI. The Table summarizes clinical trials of CMIs and the current available therapies for oHCM and the Figure summarizes the landscape of CMIs with current and potential future applications.

Several controlled studies have been performed or are underway with CMI—at a rate unparalleled in HCM in over 4 decades. The more mature trials to date have focused on symptomatic patients with oHCM. Mavacamten, the first‐in‐class CMI, has been employed in the phase 2, open label PIONEER‐HCM trial (Pilot Study Evaluating MYK‐461 in Subjects with Symptomatic Hypertrophic Cardiomyopathy and Left Ventricular Outflow Tract Obstruction). The study included 2 oHCM cohorts: cohort A (mavacamten 10–20 mg/day without background medical therapy) and cohort B (mavacamten 2–5 mg/day allowing concomitant BB administration). Mavacamten was highly effective in reducing LVOT gradient and improved pVO_2_, causing minor decreases in LVEF at higher plasma concentration.^14^ These findings led to the phase III EXPLORER‐HCM trial (Clinical Study to Evaluate Mavacamten [MYK‐461] in Adults with Symptomatic Obstructive Hypertrophic Cardiomyopathy), a randomized placebo‐controlled 30‐week on treatment trial of mavacamten in 251 patients with oHCM, on the background of BB and CCB (>90% of subjects).^16^ The trial met its primary endpoint in a highly significant fashion: an improvement in pVO_2_ by 3.0 mL/kg per minute without worsening in NYHA class or improvement of pVO_2_ by 1.5 mL/kg per minute and at least one NYHA class reduction was observed in 37% of patients on mavacamten versus 17% of patients on placebo (*P*<0.0001). Mavacamten was generally safe and well tolerated, with a general adverse event profile comparable to placebo. Only one sudden death occurred, in the placebo arm. A total of 7 patients on mavacamten developed systolic dysfunction, which was reversible with appropriate washout.^16^ The finding of increased incidence of atrial fibrillation on mavacamten in PIONEER‐HCM was not confirmed in EXPLORER‐HCM where atrial fibrillation incidence as a treatment emergent adverse event was 2% in the mavacamten group as compared with 3% on placebo.^15^, ^16^

The analysis of multiple pre‐specified secondary endpoints in Explorer‐HCM demonstrated marked and consistent improvement in LVOT gradient, symptomatic status and quality of life. Notably, 65% of patients on mavacamten had improvement by ≥1 NYHA class by week 30, as compared with 31% on placebo, and 50% of patients on mavacamten achieved NYHA class I as compared with 21% on placebo.^16^ These changes were associated with marked and sustained reduction in circulating levels of N‐terminal pro‐brain natriuretic peptide and high sensitivity troponin I in the active treatment arm. Of note, oHCM patients on BB treatment receiving mavacamten showed a modest increase in peak VO_2_, due to the blunted heart rate response, despite an amelioration in performance shown by the consistent improvement in minute ventilation/carbon dioxide production (VE/VCO_2_) slope—which is heart rate independent. Post‐hoc analyses are awaited to understand the characteristics and predictors of patients who did not respond to mavacamten.

Overall, mavacamten was effective, with a number needed to treat of 2, 3, and 4 at 30 weeks, respectively, for the endpoints of LVOT gradient reduction ≤50 mm Hg, improvement in NYHA by ≥1 class, and improvement to NYHA class I.^21^ While such proportion of responders may be seen to compare unfavorably with published SRT results, it must be emphasized that patients enrolled in EXPLORER‐HCM were not immediate candidates for SRT (operative referral being an exclusion criterion), and that about two‐thirds were in NYHA class 2, while only the remaining third were in class 3. In addition, the results of a prospective double‐blind placebo‐controlled, randomized trial cannot be compared with those from retrospective SRT studies, in view of inherent limitations of the latter. SRTs have never been formally tested in a rigorous controlled environment, and likely never will. However, the considerable placebo effect seen in EXPLORER‐HCM serves as an important reminder to use caution when interpreting observational as compared to controlled data. Because of these caveats, randomized controlled trials are needed, such as the ongoing VALOR‐HCM trial (A Study to Evaluate Mavacamten in Adults with Symptomatic Obstructive HCM Who Are Eligible for Septal Reduction Therapy, NCT04349072), whose primary aim is precisely that of assessing whether mavacamten may reduce or postpone the need for SRT (Figure and Table).

Quality of life analysis provided further insight into the efficacy of mavacamten in EXPLORER‐HCM. In a recently published pre‐specified sub‐analysis,^22^ the change in Kansas City Cardiomyopathy Questionnaire‐Overall Score (KCCQ‐OS) at the end of treatment was greater with mavacamten than placebo (14.9±15.8 versus 5.4±13.7; difference +9.1 [95% CI 5.5–12.8]; *P*<0.0001), with similar benefits across all KCCQ‐OS subscales. The proportion of patients with a very large benefit (KCCQ‐OS ≥20 points) was 36% in the mavacamten group versus 15% in the placebo group, with an estimated absolute difference of 21% (95% CI 8.8–33.4) and a number needed to treat of 5 (95% CI 3–11). Thus, mavacamten markedly improved symptoms, physical and social function and quality of life in patients with oHCM, to a degree surpassing that of most successful cardiovascular drugs, and resembling the effects of invasive interventions such as transcatheter aortic valve replacement.^22^

A small EXPLORER‐HCM cardiac magnetic resonance sub‐study comparing 17 treated patients versus 18 on placebo, showed significant positive remodeling over the 30‐week trial period, characterized by decreased left ventricular mass, wall thickness, and left atrial volume index. In the light of concomitant improvement in echocardiographic diastolic function and circulating biomarkers, these changes can be interpreted as beneficial and promising in terms of long‐term evolution of the disease, particularly as they were not associated with any increase in replacement or interstitial fibrosis, as measured by late gadolinium enhancement imaging and extracellular volume fraction.^23^ Notably, similar, positive signals have emerged in a small pilot study in nonobstructive HCM patients, suggesting that the benefits observed in EXPLORER‐HCM are not solely due to the marked reduction in LVOTO, but reflect direct effects on the myocardium.^24^ While mavacamten is still under review by the United States Food and Drug Administration for official registration, 2 ongoing 5‐year extension studies (NCT03496168 and NCT03723655) will shed more light on the long‐term safety and efficacy of the drug in HCM patients.

Aficamten is the second CMI undergoing clinical trials: it has a shorter life compared with mavacamten, achieves steady state within 2 weeks, and appears to have a wide therapeutic window.^25^ In the phase II randomized placebo‐controlled sequential cohort REDWOOD‐HCM (Randomized Evaluation of Dosing with CK‐3773274 in Obstructive Outflow Disease in HCM) trial, high‐dose aficamten (10–30 mg daily) had a favorable safety profile and led to 93% response rate (defined as a final resting LVOT gradient ≤30 mm Hg and Valsalva LVOT gradient ≤50 mm Hg) compared with 8% in the placebo arm.^13^ The ongoing REDWOOD‐HCM Cohort 3 of open‐label aficamten on the background of disopyramide is fully enrolled, with data expected soon (NCT04219826). Based on the data from REDWOOD‐HCM Cohorts 1 and 2, the phase III SEQUOIA‐HCM trial is set to start enrolling.

The development of ground‐breaking, disease‐specific therapies, represent a major opportunity for HCM patients, in the attempt to improve longevity, minimize morbidity, and improve quality of life. However, it would be wrong to view these novel therapeutic options as competitors to currently available treatments: they are just another arrow to our bow. As discussed earlier, providing more options ultimately means allowing patients the possibility of choosing the therapy that best suits their needs, values and circumstances. At the same time, we must be aware that early enthusiasms should be tempered by caution until we learn more about their long‐term efficacy and safety, as well as their cost‐effectiveness. Many open questions remain regarding CMI, ranging from potential benefits in non‐obstructive HCM, individual variability in response in different ethnic groups, and their potential in preventing phenotype development in HCM mutation‐carriers.

In the specific case of oHCM, it is early to say if and how much CMI will impact current practice including SRT referrals. A hypothetical transition of invasive interventions from a default strategy to a therapy for selected non‐responders to CMI might represent a major advancement, certainly welcomed by patients. Beyond the laudable goal of improving hemodynamic parameters and symptoms, the Holy Grail lies in the possibility that CMI may effectively address the core mechanism of HCM, influence its substrates and prevent its natural course. Mapping this uncharted territory will require intense and prolonged efforts. Nevertheless, favorable clinical evidence is rapidly accumulating with CMI as well as its functional counterpart omecamtiv mecarbil—a myosin activator effective in heart failure with reduced ejection fraction.^26^ With additional trials underway and molecules in development, it is fair to say that the newborn strategy of myosin modulation is here to stay in cardiovascular medicine.

## Disclosures

Masri has received research grants from Pfizer, Ionis, Akcea, Ultromics and the Wheeler Foundation, fees (honoraria or consulting) from Eidos, Pfizer, Ionis, Alnylam, Cytokinetics, Bristol Meier Squibb, Tenaya, and Attralus, and served or currently serving as PI on EXPLORER‐HCM, PIONEER‐OLE, MAVA‐LTE, VALOR‐HCM, REDWOOD‐HCM, REDWOOD‐OLE, and SEQUOIA‐HCM trials. Olivotto has received grants from Bristol Meier Squibb, Cytokinetics, Amicus, Genzyme, Shire, Bayer, Boston Scientific, Menarini International, and fees (honoraria or consulting) from Bristol Meier Squibb, Cytokinetics, Amicus, Genzyme, Shire, Boston Scientific, and served or currently serving as PI on EXPLORER‐HCM, MAVA‐LTE, REDWOOD‐HCM, REDWOOD‐OLE, and SEQUOIA‐HCM trial.

**Response to Masri and Olivotto**

Barry J. Maron, MD; Martin S. Maron, MD; Mark V. Sherrid, MD; Ethan J. Rowin, MD

We welcome therapeutic advances that benefit HCM patients. Nevertheless, we do not agree with some arguments presented regarding myosin inhibitors.

The authors have not completely described current management of HCM, the arena mavacamten would build upon. Surgical myectomy and alcohol ablation are highly effective at reversing heart failure in most obstructive patients with low risk in expert centers. Performed as a one‐time procedure, myectomy eliminates need for long‐term medical therapy, inevitably associated with high cost typical of novel drug treatments. Indeed, the maturation of myectomy/ablation has been partly responsible for reduced HCM mortality (to only 0.5%/year).

Highly favorable Real world outcome data in thousands of HCM patients over decades does not align with Authors’ assertion that somehow the present “status quo” is unacceptable, fails to “empower patients” or personalize care. Claims that myosin inhibitor drugs address the “core (molecular) mechanism of disease” is an attractive hypothesis but without substantiating evidence. Mavacamten actions beyond its negative inotropic gradient reduction are speculative at present.

The EXPLORER‐HCM data clearly document that mavacamten can reduce LV outflow gradients. However, there are two other areas that did not receive proper attention. First, most patients (two‐thirds) did not meet the short‐term pre‐defined primary end‐point of subjectively improved symptoms and/or modest increases in peak VO_2_, suggesting that many of these patients with limited clinical benefit may be (or soon become) potential candidates for myectomy/ablation. Second, risk for systolic dysfunction is understated, odd considering the importance FDA has already placed on this safety issue. If unrecognized in practice, reduced EF <50% could be associated with heart failure symptoms, underscoring importance of long‐term vigilance with frequent echocardiography.

Myosin‐inhibitors will have a role in management of symptomatic obstructive HCM. However, based on our extensive experience with HCM, prudent perspectives regarding mavacamten (rather than unbridled enthusiasm) would be in the best interests of patients.

**Table:** Randomized Trials of Current Available Therapies and CMIs in oHCM

Intervention	Trial name and NCT number	Design	Intervention and treatment duration	Number of subjects	Summary of main findings
Randomized trials of standard of care					
Beta blockers and calcium channel blockers	Gilligan et al^9^	Double‐blind, placebo‐controlled crossover trial	Nadolol Verapamil Placebo 4 wk each	18	PVO_2_ not statistically different between groups. Peak exercise workload was reduced by ≥10 W in 81% on nadolol and 25% on verapamil as compared with placebo
	Dybro et al^12^	Double‐blind, placebo‐controlled crossover trial	Metoprolol Placebo 2 wk each	29	Resting LVOT gradient on metoprolol was 25 mm Hg (15–58) vs 72 mm Hg (28–87) on placebo. Peak exercise LVOT gradient was 28 mm Hg (18–40) vs 62 (31–113) on placebo. During metoprolol treatment, 14% of patients were in NYHA functional class III or higher compared with 38% of patients receiving placebo (*P*<0.01). KCCQ‐OSS during metoprolol treatment was 76.2±16.2 vs 73.8±19.5 on placebo; *P*=0.039. Measures of exercise capacity, peak oxygen consumption, and N‐terminal pro–B‐type natriuretic peptide did not differ between metoprolol and placebo
Disopyramide	None				
ASA	None				
Myectomy	None				
Ventricular pacing with short atrioventricular‐delay	PIC trial^11^	Double‐blind, randomized crossover trial	Pacing vs backup pacing 12 wk each	83	Peak LVOT gradient improved from 59±36 to 30±25 mm Hg with active pacing and Improvement in NYHA class (2.4–1.7)
	M‐Pathy trial^10^	Double‐blind, randomized crossover trial, followed by open‐label 6‐mo pacing trial	Pacing vs backup pacing 12 wk each	48	Peak LVOT gradient improved from 82±32 to 48±32 mm Hg. No improvement in pVO_2_ or functional measures in the randomized period
Randomized trials of CMIs					
Mavacamten	PIONEER‐HCM Cohort A^14^ NCT02842242	Open‐label mavacamten	Mavacamten 10–15 mg and no background medical therapy 12 wk	11	Improvement in pVO_2_ (+3.5 mL/kg per min, 95% CI 1.2–5.9) and reduction of peak‐exercise LVOT gradient by 90 mm Hg (95% CI −138 to −41). Decreased LVEF occurred in 3 subjects with recovery
	PIONEER‐HCM Cohort B^14^ NCT02842242	Open‐label mavacamten in patients with NYHA class II–III	Mavacamten 2–5 mg daily with beta blockers 12 wk	10	Improvement in pVO_2_ (+1.7 mL/kg per min, 95% CI 0.0–3.3) and reduction of peak‐exercise LVOT gradient by 25 mm Hg (95% CI −47 to −3.0). None had decreased LVEF
	PIONEER‐OLE NCT03496168	Open‐label mavacamten	Mavacamten 2.5–15 mg daily 260 wk	12	Ongoing. Data presented in meeting proceedings but no peer‐reviewed publication
	EXPLORER‐HCM^16^ NCT03470545	Randomized placebo‐controlled double‐blind trial in patients with NYHA class II–III	Mavacamten 2.5–15 mg 30 wk	251	Mavacamten met the co‐primary endpoint (improvement in pVO_2_ by 3.0 mL/kg per min without worsening in NYHA class or improvement of pVO_2_ by 1.5 mL/kg per min and at least one NYHA class reduction) in 37% of patients vs 17% of patients on placebo. LVEF to ≤50% in 7 patients and with recovery of LVEF in all
	MAVA‐LTE NCT03723655	Open‐label but with triple masking of dose (patients, provider and investigator).	Mavacamten (follows similar dosing as EXPLORER‐HCM and MEVERICK‐HCM) 252 wk	310	Ongoing. Data presented in meeting proceedings but no peer‐reviewed publication
	VALOR‐HCM NCT04349072	Randomized, double‐blind, placebo‐controlled trial in patients referred to SRT	Mavacamten 2.5–15 mg 32 wk (primary outcome at week 16)	100	Fully enrolled and results expected in 2022
Aficamten	REDWOOD‐HCM Cohort 1 and 2 NCT04219826	Randomized, double‐blind, placebo‐controlled trial in patients with NYHA class II–III	Aficamten: Cohort 1 (5–15 mg) Cohort 2 (10–30 mg) 10 wk	41	Ongoing. Data presented in meeting proceedings but no peer‐reviewed publication
	REDWOOD‐HCM Cohort 3 NCT04219826	Open‐label aficamten in patients on disopyramide in patients with NYHA class II–III	Aficamten 5–15 mg 10 wk	13	In active follow up
	REDWOOD‐OLE NCT04848506	Open‐label trial	Aficamten 5 y	54	In active follow up
	SEQUOIA‐HCM	Randomized, double‐blind, placebo‐controlled trial in patients with NYHA class II–III	Aficamten 5–20 mg 24 wk on treatment	270	Has not started enrolling

ASA indicates alcohol septal ablation; CMI, cardiac myosin inhibitors; KCCQ‐OSS, Kansas City Cardiomyopathy Questionnaire‐Overall Summary Score; LVEF, left ventricular ejection fraction; LVOT, left‐ventricular outflow tract; NCT, national clinical trial number; nHCM, non‐obstructive hypertrophic cardiomyopathy; NYHA, New York Heart Association; oHCM, obstructive hypertrophic cardiomyopathy; pVO_2_, peak oxygen consumption; SRT, septal reduction therapy; and TBD, to be determined.

**Figure:**
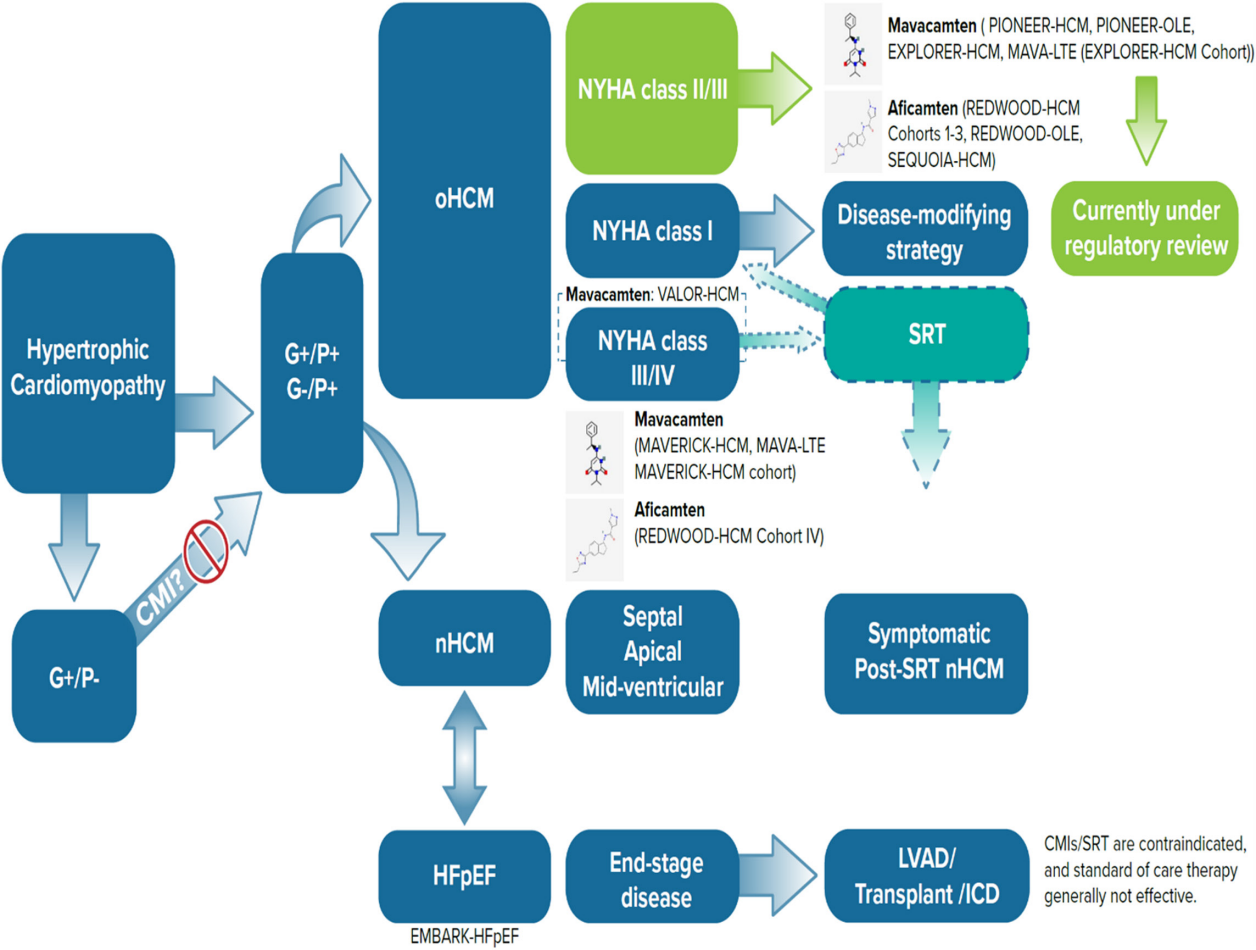
The landscape of current and future applications of cardiac myosin inhibitors (CMI) for the whole spectrum of hypertrophic cardiomyopathy. Currently, the most mature application for CMI is symptomatic oHCM with NYHA class II/III, where mavacamten is under regulatory review (green boxes). Other possible future applications of CMIs include disease modifying strategies in asymptomatic individuals (de‐novo and post‐SRT), in oHCM who are referred for SRT, and in nHCM with septal, apical, mid‐ventricular, and post‐SRT phenotypes. Patients with heart failure with preserved ejection fraction also represent a future target for CMIs given their mechanism of action. Finally, in the minority of patients who present or progress to end‐stage disease (defined as a left ventricular ejection fraction ≤50%), CMIs and SRT are contraindicated and/or not beneficial, and standard of care therapies are not typically effective. In these scenarios, advanced heart failure therapies are required. CMs indicates cardiac myosin inhibitors; G−, genotype negative; G+, genotype positive; HFpEF, heart failure with preserved ejection fraction; ICD, internal cardioverter defibrillator; LVAD, left ventricular assist device; nHCM, non‐obstructive hypertrophic cardiomyopathy; NYHA, New Yok Heart Association; oHCM, obstructive hypertrophic cardiomyopathy; P−, phenotype negative; P+, phenotype positive; and SRT, septal reduction therapies.
